# Fetal Programming of Renal Dysfunction and High Blood Pressure by Chronodisruption

**DOI:** 10.3389/fendo.2019.00362

**Published:** 2019-06-06

**Authors:** Natalia Mendez, Claudia Torres-Farfan, Esteban Salazar, Pía Bascur, Carla Bastidas, Karina Vergara, Carlos Spichiger, Diego Halabi, Carlos P. Vio, Hans G. Richter

**Affiliations:** ^1^Laboratory of Developmental Chronobiology, Institute of Anatomy, Histology, and Pathology, Faculty of Medicine, Universidad Austral de Chile, Valdivia, Chile; ^2^Centro Interdisciplinario de Estudios del Sistema Nervioso, Universidad Austral de Chile, Valdivia, Chile; ^3^Faculty of Sciences, Institute of Biochemistry and Microbiology, Universidad Austral de Chile, Valdivia, Chile; ^4^Faculty of Medicine, School of Dentistry, Universidad Austral de Chile, Valdivia, Chile; ^5^Center of Aging and Regeneration CARE, Department of Physiology, Pontificia Universidad Católica de Chile, Valdivia, Chile; ^6^Facultad de Medicina y Ciencia, Universidad San Sebastian, Santiago, Chile

**Keywords:** blood pressure, chronodisruption, kidney, fetal programming, high-salt diet, transcriptome landscape, DOHaD

## Abstract

Adverse prenatal conditions are known to impose significant trade-offs impinging on health and disease balance during adult life. Among several deleterious factors associated with complicated pregnancy, alteration of the gestational photoperiod remains largely unknown. Previously, we reported that prenatal manipulation of the photoperiod has adverse effects on the mother, fetus, and adult offspring; including cardiac hypertrophy. Here, we investigated whether chronic photoperiod shifting (CPS) during gestation may program adult renal function and blood pressure regulation. To this end, pregnant rats were subjected to CPS throughout pregnancy to evaluate the renal effects on the fetus and adult offspring. In the kidney at 18 days of gestation, both clock and clock-controlled gene expression did not display a daily pattern, although there were recurrent weaves of transcriptional activity along the 24 h in the control group. Using DNA microarray, significant differential expression was found for 1,703 transcripts in CPS relative to control fetal kidney (835 up-regulated and 868 down-regulated). Functional genomics assessment revealed alteration of diverse gene networks in the CPS fetal kidney, including regulation of transcription, aldosterone-regulated Na+ reabsorption and connective tissue differentiation. In adult offspring at 90 days of age, circulating proinflammatory cytokines IL-1β and IL-6 were increased under CPS conditions. In these individuals, CPS did not modify kidney clock gene expression but had effects on different genes with specific functions in the nephron. Next, we evaluated several renal markers and the response of blood pressure to 4%NaCl in the diet for 4 weeks (i.e., at 150 days of age). CPS animals displayed elevated systolic blood pressure in basal conditions that remained elevated in response to 4%NaCl, relative to control conditions. At this age, CPS modified the expression of *Nhe3, Ncc, Atp1a1, Nr3c1* (glucocorticoid receptor), and *Nr3c2* (mineralocorticoid receptor); while *Nkcc, Col3A1*, and *Opn* were modified in the CPS 4%+NaCl group. Furthermore, CPS decreased protein expression of Kallikrein and COX-2, both involved in sodium handling. In conclusion, gestational chronodisruption programs kidney dysfunction at different levels, conceivably underlying the prehypertensive phenotype observed in the adult CPS offspring.

## Introduction

Modern society relies on artificial light for a substantial part of the 24 h; however, excessive exposure to artificial light may have negative impacts on human health (1, 2). It has been reported that exposure to artificial light at night is linked with adverse effects on several physiological systems due to disruption of the local biological clocks and, consequently, misalignment of the entire circadian system (3, 4). The circadian system includes a master clock residing in the suprachiasmatic nucleus (SCN) of the brain and peripheral clocks present in nearly every tissue and organ (5). The SCN synchronizes peripheral clocks through neural and hormonal signals (a key role being played by melatonin), therefore aligning the whole circadian system to the external light-dark cycle. At the cellular level, biological clocks rely on a set of molecular players called *clock genes* (i.e., *Clock, Bmal1, Cry1/2, Per1/2*, and *Rev-erba*) which in turn regulate the expression of specific proteins (6). These clockwork elements establish an interlocking transcription-translation feedback loop generating circadian oscillations of mRNAs and proteins providing the frame for the internal temporal order of all physiological processes, including blood pressure (BP), metabolism and renal function, among others (5).

The integrity of the circadian system is critical for normal function of different physiological systems; in fact, alteration of the circadian system or “chronodisruption” has been demonstrated to have numerous adverse consequences on health (7–9). An essential characteristic of the circadian system is its ability to be synchronized by external time cues, with the light/dark cycle being one the most important signals (3, 4). Shift work is a condition that chronically impairs adequate function of biological clocks, favoring multiple pathological processes like cancer, metabolic and cardiovascular disorders (4, 9–11). Many of these processes could be related with a low-grade inflammation state, as suggested by the up regulation of TNF-α, IL-1β, and IL-6 in various tissues from rat and other animal models of chronodisruption by sleep deprivation (12, 13).

Detrimental reproductive effects have been observed in shift worker women, such as alteration of the menstrual cycle, higher incidence of miscarriage and impaired fetal development (10, 14–17). Conditions negatively influencing fetal growth and development exert a long-term impact on health that persists across life, concept known as fetal programming of adult disease (18). Indeed, epidemiological and experimental studies show that impaired fetal growth is a strong predictor of obesity, hypertension, and diabetes in adulthood and therefore it may program enhanced vulnerability to secondary insults (19, 20). Despite this evidence, disruption of the maternal circadian system during gestation or “gestational chronodisruption” has been little explored.

Epidemiological studies suggest that the origins of high blood pressure may be tracked back to fetal life (19, 21). In turn, the kidney is considered an essential organ controlling blood pressure, since the onset of hypertension is usually associated with some disturbance in renal function (22, 23). Currently, there is no evidence linking gestational chronodisruption with fetal programming of renal dysfunction and blood pressure, although a relationship between the circadian system and renal physiology has been described (24, 25). Renal function displays both molecular and physiological circadian oscillations (24). Some genes like aquaporin water channels, vasopressin receptor, *Nhe3* sodium-proton exchanger, serum-induced and glucocorticoid-induced kinase 1 (*Sgk1*) among others, are controlled by circadian mechanisms at the transcriptional and posttranslational level (26). Changes in the expression of some of these genes may lead to adjustment in the filtration area, bringing about changes in renal tubular function, in turn resulting in changes in the expression of transporters that might induce salt-sensitive hypertension (27). In addition, there is evidence of oscillatory capacity of an early molecular clock in the kidney, characterized by circadian expression of key clock and clock-controlled genes already at 20 days of gestation in the rat (28). The early rhythmic expression of clock and clock-controlled genes in the kidney suggests that this peripheral clock may be influenced by maternal circadian cues, underscoring the importance of the integrity of the maternal circadian system during development (see Discussion for details). We hypothesized that chronic photoperiod shifting during gestation (CPS; up to 85% of gestation) triggers changes in the fetal kidney with possible consequences on postnatal renal function and blood pressure regulation. Therefore, here we studied the effects of circadian disruption during gestation on gene expression in the fetal and adult offspring's kidney, together with several renal markers and the response of blood pressure to prolonged 4%NaCl treatment in the adult offspring.

## Materials and Methods

### Ethical Approval

The protocols were approved by the Bioethics Commission from the Universidad Austral de Chile (CBA: 221/2015; 297/2017). Animal handling was performed following the guidelines for the care and use of Laboratory Animals of the Institute for Laboratory Animals Research of the National Research Council.

### Animals and Experimental Procedures

Sprague-Dawley rats were obtained from Charles River (CRL International Inc., Kingston NY) and housed in our facility under standard photoperiod (12:12 h light-dark cycle), lights on at 07:00 AM; ~400 lx at the head level; with controlled temperature (20 ± 2°C), food and water *ad libitum*. At 70–80 days of age (250–300 g) the females were mated and day zero of gestation (E0) was determined by positive sperms in the vaginal smears. Pregnant females were separated randomly into two groups: LD group, pregnant females kept under 12:12 h throughout gestation and CPS group (chronic photoperiod shifting) corresponding to pregnant females kept under a rotation scheme of photoperiod used recently by us. Briefly, pregnant females were exposed to lighting schedule manipulation every 3–4 days, reversing completely the photoperiod (see Supplementary Figure S1). The photoperiod reversal occurred on the night of day 0 of gestation so that rather than lights going off at 07:00 PM they remained on until 07:00 AM of day 2. At 18 days of gestation, the mothers returned to a normal 24 h photoperiod (12:12 h light-dark cycle, lights on at 07:00 AM) and continued under this photoperiod schedule thereafter (29). No signals of stress were observed in the pregnant rats exposed to CPS since we did not observe hair loss, stereotyped movements or altered water/food intake during gestation (29, 30). On the other hand, no fetal reabsorptions were observed, since the number of fetuses per mother was similar in both experimental groups (29).

In the study, three cohorts of animals were used to assess the effects of CPS on the fetal kidney, daily gene expression in the adult kidney at 90 days of age, and blood pressure response to 4%NaCl in the adult offspring.

### Study in Fetal Kidney

We obtained fetal tissues from pregnant females of each experimental group (LD and CPS). The dams were euthanized every 4-h around the clock beginning at 08:00 h on E18 and ending at 08:00 h on E19 (*n* = 5 per clock time; LD, *n* = 30; CPS, *n* = 29 mothers). Pregnant rats were anesthetized (isoflurane 3.0–4.0%; Baxter Laboratories), a midline incision was done, and an overdose of sodium thiopental was administrated (150 mg/kg; Vetpharma). Fetuses were euthanized by spinal transection, weighed and the kidneys were immediately dissected out under sterile conditions. The fetal tissues collected were stored at −20°C in RNAlater® for the study of daily expression of genes related with clock machinery (*Bmal1, Per1, Per2*, and *Dbp*), and genes involved in renal function *Nhe3*, α*Enac, Sgk1*, and glucocorticoid receptor (*Nr3c1*).

At E19 we evaluated the global fetal transcriptome by microarray analysis (non-sexed pool of fetal kidneys coming from 6 mothers kept under CPS and LD conditions; sampled at 08:00 h). RNA was extracted using the “SV Total RNA Isolation System” according to the manufacturer's instructions (Promega, Madison, WI). RNA quality was assessed with the Agilent 2,100 Bioanalyzer and the associated RNA 6,000 Nano and Pico LabChip kits (Agilent Technologies, Palo Alto, CA). RNA samples with RNA integrity score 7 or higher were used for further microarray analysis. Sample processing and microarray hybridization were performed by an external dedicated Core Facility, KFB - Center of Excellence for Fluorescent Bioanalytics (Regensburg, Germany; http://www.kfb-regensburg.de). All steps of microarray analysis were carried out as previously described (30), using Affymetrix Rat Gene 2.1 ST GeneChip arrays (containing 700,000 probe sets representing 28,000 rat genes). All array data were normalized using Robust Multiarray Average (RMA). RMA utilizes the probe set annotation provided by Affymetrix to identify genes directly from the CEL files. Genes differentially expressed between experimental groups were determined with the Transcriptome Analysis Console software (TAC, Affymetrix, Santa Clara, CA) using a *p*-value < 0.05 as threshold (one-way between-subject unpaired ANOVA) (30). The same RNA was used for validation by RT-qPCR. Ingenuity Pathway Analysis (IPA) and DAVID 6.7 were used for functional genomics analysis of differentially expressed genes as described previously (31, 32). The full data set for global transcription analysis by microarray is available at the following NCBI online repository link (www.ncbi.nlm.nih.gov/geo/query/acc.cgi?acc=GSE130744).

### Study in Adult Male Offspring

To evaluate the long-term effects of CPS, we used two parallel cohorts of animals from each pregnancy condition (LD and CPS). After delivery, the CPS litters and their mothers returned to standard photoperiod (12:12 h); from this point onwards, the animals grew up in the same conditions than LD litters. The pups were weaned at 21 days-old; the males of each condition were housed in pairs (brothers together in standard cages 48 × 27 × 20 cm) until the age of study at 90 days-old. In the adult male offspring, we evaluated the effects of gestational chronodisruption on gene expression in the kidney as well as blood pressure response to 4%NaCl in the diet. Adult female tissues from each condition were stored in our tissue bank for further studies. To measure circulating cytokines in adult offspring at 90 days of age, blood samples were collected at 10:00 h AM (LD *n* = 4 and CPS *n* = 5). A group of selected pro- and anti-inflammatory cytokines were evaluated in the adult plasma, using Milliplex RECYTMAG-65K and RECYMAG65K27PMX kits.

### Study of Oscillatory Gene Expression in the Adult Offspring's Kidney

At 90 days of age, male offspring animals were euthanized every 4-h around the clock (*n* = 4–5 individuals per clock time: LD *n* = 28 males; CPS *n* = 26 males), starting at 08:00 h and ending at 04:00 h. To avoid litter effects each batch contained animals from different mothers; thus, no siblings were used in the same clock time batch. The males were euthanized as described before and their kidneys were collected, weighed, and stored in RNAlater (Ambion Inc. Austin, TX, USA) until RNA extraction.

### Quantitative Real-Time PCR

Quantitative RT-qPCR was used to evaluate mRNA expression. In the kidney, we studied the expression of a comprehensive set of clock and clock-controlled genes (*Bmal1, Per-1, Per-2, Nhe3, Nkcc, Ncc, Romk*, α*Enac, Aqp2, Atp1a1, At1r, Nr3c1, Nr3c2 Renin, Avpr2, Sgk1, Eca, Cox-2*, and *kallikrein*). Total RNA was extracted using the SV total RNA isolation system (Promega) according to the manufacturer's instructions. About 2.0 μg of total RNA were reverse transcribed using random primers (Promega) and Moloney murine leukemia virus reverse transcriptase (Invitrogen Corp). RT-qPCR was performed using primers described in Table 1 and KAPA SYBR FAST quantitative PCR master mix (Kapa Biosystems, Inc). The quantitative PCR was carried out in a Rotor-GeneQ real-time platform (QIAGEN). Relative amounts of all mRNAs were calculated by the comparative ΔΔCt method (33). To validate the quantification using the ΔΔCt method in each gene analyzed, a dilution curve (range 10–100 ng input total RNA) was performed in the calibrator (a kidney pool from LD condition). The Ct obtained vs. RNA concentration was plotted and the slope (m) of the lineal regression was calculated, with the efficiency (*E*) in each curve for each gene being determined as *E* = 10^−(1/m)^. Efficiencies were between 2.01 and 2.10 (equivalent 101–110%, respectively). The first ΔCt is the difference in the sample of the Ct values between the gene of interest and its respective housekeeping gene (*Hprt or Gadph*), while the second ΔCt is the difference in the calibrator sample between the gene of interest and its respective housekeeping. The sample and the calibrator were assayed simultaneously. All the samples were amplified in duplicate in at least three mRNA concentrations (range 10–100 ng). A melting curve analysis was performed on each sample after the final cycle to ensure that a single product was obtained, and agarose gel electrophoresis confirmed that the single PCR product was of the expected size. Since we found that *Gadph* expression is higher in the fetus vs. the expression of the gene selected, we used *Hprt* to normalize gene expression in the fetus measurement. In contrast, in the adult kidney we used *Gadph* to normalize gene expression.

**Table 1:** Primers used for RT-qPCR studies.

**Symbol**	**Gene bank**	**Primer sequence (5′->3′)** **Fw/Rv**	**Amplicon length (bp)**	**Annealing temperature (°C)**
*Nr3c2*	NM_013131.1	CCAAAGGCTACCACAGTCTC/ TCCCAGACCGACTATTGTCT	240	60
*Nr3c1*	NM_012576.2	ACAGCTCACCCCTACCTTGGT/ CTTGACGCCCACCTAACATGT	134	60
*Col3a1*	NM_032085.1	CTTCTCACCCTGCTTCACCC/ GGGCAGTCTAGTGGCTCATC	194	60
*Kall*	NM_031523.1	GCATCACACCTGACGGATTG/ GGCCTCCTGAGTCACCCTTG	171	60
*Opn*	NM_012881.2	AGTGGTTTGCTTTTGCCTGTT/ TCAGCCAAGTGGCTACAGCAT	122	60
*At1r*	NM_031009.2	CAAAAGGAGATGGGAGGTCA/ TGACAAGCAGTTTGGCTTTG	254	60
*Tgfb*	NM_021578.2	GCCAGATCCTGTCCAAACTAA/ TTGTTGCGGTCCACCATTA	202	60
*Eca*	NM_012544.1	AACACGGCTCGTGCAGAAG/ CCTGCTGTGGTTCCAGGTACA	79	60
*Cox-2*	NM_017232.3	TGTATGCTACCATCTGGCTTCGG/ GTTTGGAACAGTCGCTCGTCATC	94	60
*Atp1a1*	NM_012504.1	CCCAAAACGGACAAACT/ GCACTACCACGATACTGAC	274	60
*Romk*	NM_017023.2	CACTGTGCCATGTGCCTCTA/ ATCTGGGTGTCGTCCGTTTC	113	60
*Ncc*	NM_019345.3	GAGAACGGCACACCCATTG/ GACAAGAAAGAACAGCACCTGG	135	60
*Nkcc2*	NM_001270618.1	GCAGGGATCTTTTCGGCAAC/ CTCTCAGGGGCTCGTTGTTT	148	60
*Aqp2*	NM_012909.2	TCACTGGGTCTTCTGGATCG/ CGTTCCTCCCAGTCGGTGT	147	60
*Hsd11b2*	NM_017081.2	CGCGAATGTATGGAGGTG/ CAGTTGCTTGCGCTTCTC	291	60
*Nhe3*	NM_012654.1	ATGGAGAATCTGGCACAC/ TGGCACCCTGGATAGGAT	213	60
*Renin*	NM_012642.4	GCTACATGGAGAATGGGACTGAA/ ACCACATCTTGGCTGAGGAAAC	79	60
*Per2*	NM 031678	CACCCTGAAAAGAAAGTGCGA/ CAACGCCAAGGAGCTCAAGT	148	62
*Bmal1*	NM024362.2	CCGATGACGAACTGAAACACCT/ TGCAGTGTCCGAGGAAGATAGC	215	64
*Hprt*	NM_012583.2	CCATCACATTGTGGCCCTCT/ TATGTCCCCCGTTGACTGGT	166	60
*Gapdh*	NM_017008.4	CTCCCTCAAGATTGTCAGCA/ CCACAGTCTTCTGAGTGGCA	140	60

### Blood Pressure Response to 4%NaCl

In the second cohort of animals we measured Systolic Blood Pressure (SBP) in adult offspring from each experimental protocol (LD *n* = 8 and CPS *n* = 8). The animals were trained to the procedure for at least 10 min for seven days. Then, we evaluated the SBP in conscious animal using an ultrasonic Doppler flow detector 811-B (PARKS Electronics) placed in the tail; and obtained the mean of five records at 12:00 and 20:00 h, the criteria for clock time selection was based in our previous assessment of SBP daily rhythm (29). At 90 days of age, we registered SBP in basal conditions and its response to high sodium diet (4%NaCl) for 4 weeks (LD+4%NaCl and CPS+4%NaCl; *n* = 8, respectively). In parallel, we registered eight adults LD and CPS that received a control diet for 4 weeks (0.26% NaCl; Lab diet Prolab RMH3000).

### Markers of Renal Injury and Function

After 4 weeks under 4%NaCl diet, the animals were placed in metabolic cages to collect urine; which was centrifuged 10 min at 4,000 rpm (22°C) and subsequently stored for analysis of protein, albumin, creatinine, and electrolytes (concentrations are expressed relative to the final urine volume collected in 16-h; from 17:00 h until 09:00 h).

Besides, urinary pathological markers like kidney injury molecule-1 (KIM-1), clusterin (Clu), albumin (Alb), total protein, β2-microglobulin (β2-M), cystatin C (Cys-C), osteopontin (Opn), calbindin-D28 (Cal), glutathione-S-transferases (GST), tissue inhibitor of metalloproteinases (TIMP-1), vascular endothelial growth factor (VEGF), EGF, Lipocalin, calbindin, IP-10 and α-1-Acid Glycoprotein (AGP) were determined using Millipore Milliplex Rat Mag Kidney Toxicity Panels 1 and 2 on the Luminex® xMAP® platform (EMD Millipore Corporation, Billerica, MA, USA), following the manufacturer's instructions. Intra- and inter-assay variabilities were lower than 10% and 15%, respectively. Finally, at 150 days of age, the kidneys were collected and weighted, and used for the study of gene expression by RT-qPCR (genes listed above) and Kallikrein and COX-2 protein expression by immunohistochemistry.

### Tissue Processing and Immunohistochemical Analysis

Kidney sections were fixed in Bouin's solution and embedded in Paraplast Plus. The analysis was carried out in 5 μm thick sections. The dewaxed and washed sections were incubated for 5 min with Harris hematoxylin, washed with water (10 min) and with a 1% sodium borate solution (2 min); then, they were incubated with an aqueous solution of eosin for 5 min. These sections were used to study the expression of Kallikrein and COX-2 through immunohistochemistry. Briefly, the tissue was dewaxed, rehydrated, rinsed in 0.05 mol/L Tris-phosphate-saline buffer pH 7.6, and then incubated overnight at 22°C with the primary antibody Kallikrein: Anti-KK, developed in rabbit (1:2,500); Renal Physiology Laboratory; Vio, CP); and Anti-COX-2, developed in goat (1:1,500); Santa Cruz sc-1747), followed with appropriate secondary antibody and PAP complex (MP Biomedicals, Inc., Aurora, OH) applied for 30 min. Peroxidase activity assay was carried out with 0.1% (w/v) 3,3′-diaminobenzidine and 0.03% (v/v) hydrogen peroxide. Tissue sections were observed on a Nikon Eclipse E600 microscope, and non-overlapping images were photographed with a Nikon DS-Ri1 digital camera. Randomly selected sections were used for each animal and four images per section were taken. The stained Kallikrein and COX-2 areas in each image were quantified utilizing computer-assisted image analysis software (Simple PCI, Hamamatsu). Morphometric analysis of tissue was made in a blind observer manner.

### Statistical Analysis

To determine differences between LD and CPS conditions in fetal gene expression we used the sine wave with non-zero baseline (Y = Amplitude^*^sin ([2^*^pi^*^X/Wavelength]+PhaseShift) + Baseline) when wavelength was equal to 8 h. To assess 24 h changes in relative gene expression in adult offspring, mean data for each clock time was fitted to a theoretical cosine function to calculate hour of acrophases and correlation coefficients and 24-h mean expression were calculated for the group. To determine clock time changes within a group we used one-way ANOVA and Tukey test, to compare data between LD and CPS groups we used two-way ANOVA with Bonferroni multiple comparison tests. Mean 24 h values of relative gene expression, between LD and CPS kidneys were compared by Student's *t*-tests. To determine differences between LD, CPS, LD+4%NaCl and CPS+4%NaCl we used Kruskal-Wallis test and Two-stage step-up method of Benjamini, Krieguer and Yekutieli as *post hoc* test. Analyzes were performed using Graph Pad Prism Software V7.0, USA. Differences with *P* < 0.05 were considered statistically significant. All the tables contain the information of the statistical methods used. Data are presented as mean ± SEM.

## Results

### Gene Expression in Fetal Kidney

**Figure 1:**
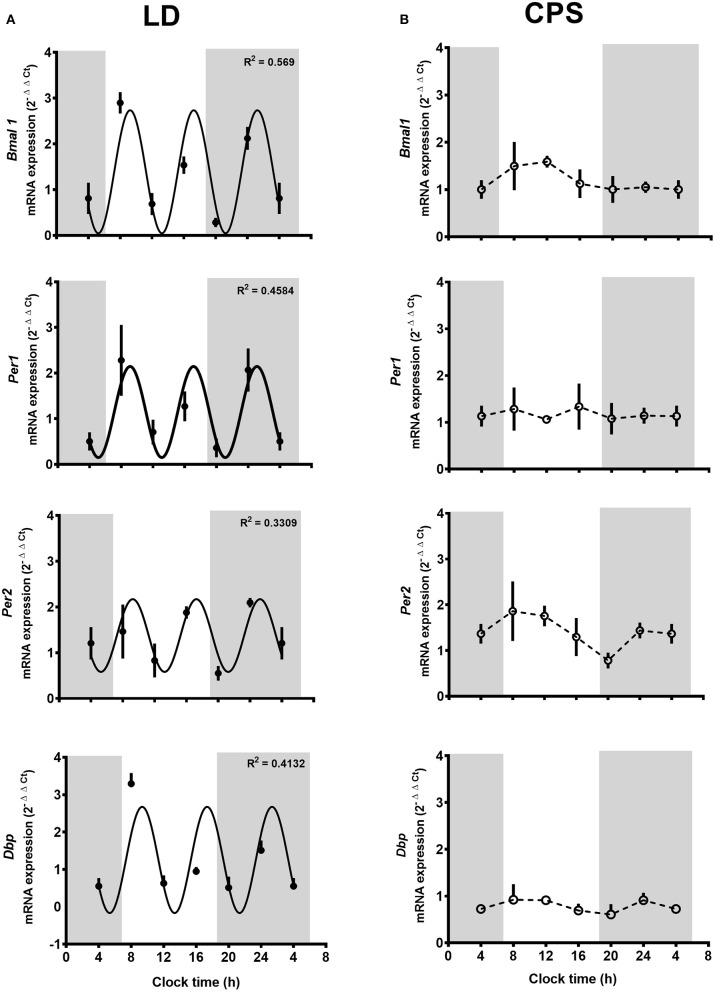
**(A,B)** Clock gene expression in the fetal kidney at 18 days of gestation. Fetal kidneys were collected every 4 h around the clock (*n* = 5/clock time; LD, *n* = 30 mothers; CPS, *n* = 29 mothers), starting at 08:00 h on day 18 and ending at 08:00 h on day 19 of gestation. Data are mean ± SEM. Solid lines represent the theoretical 8 h cosinor function in LD (*Bmal1*: R^2^ 0.569, df 31; *Per1*: R^2^ 0.4584, df 24; *Per2* R^2^ 0.3309 df 22 NS; *Dbp*: R^2^ 0.4132; df 27); while dotted lines represent CPS kidney gene expression (*R*^2^ < 0.01 for all the genes evaluated). Gray bars indicate the clock time of lights off. *P* < 0.05 Pearson's r.

**Figure 2:**
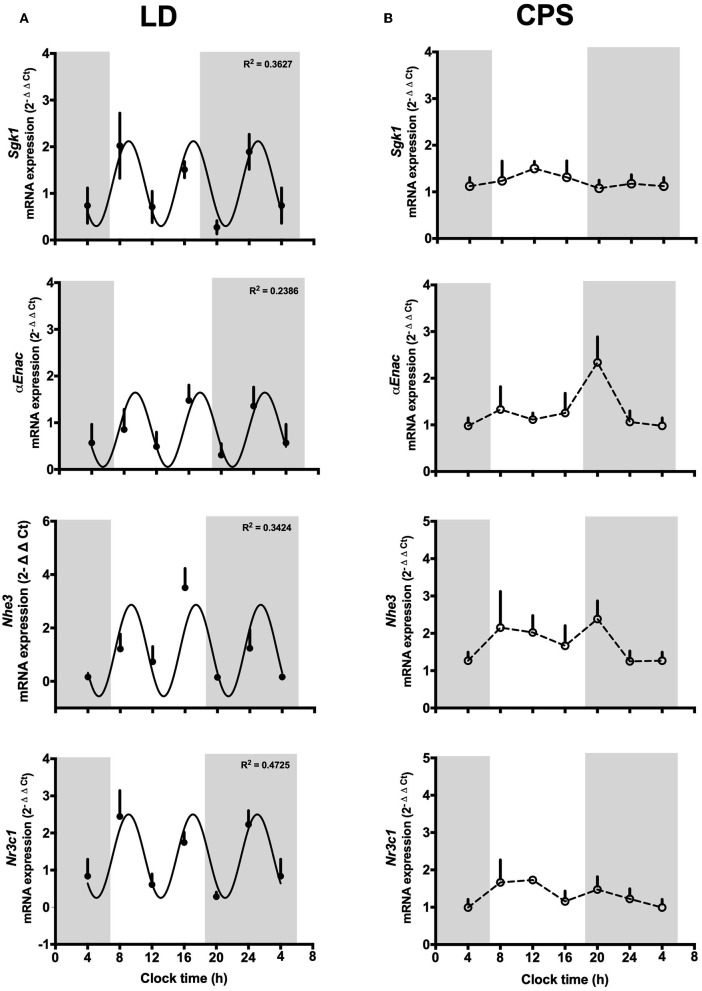
**(A,B)** Fetal expression of genes related with renal function at 18 days of gestation. Fetal kidneys were collected every 4 h around the clock (*n* = 5/clock time; LD, *n* = 30 mothers; CPS, *n* = 29 mothers), starting at 08:00 h on day 18 and ending at 08:00 h on day 19 of gestation. Data are mean ± SEM. Solid lines represent the theoretical 8 h cosinor function in LD (*SGk1*: R^2^ 0.3627, df 27; α*ENaC*: R^2^ 0.2386, df 23 NS; *Nhe3*: R^2^ 0.3424, df 23 NS; *Nr3c1*: R^2^ 0.4725, df 26) and dotted lines represent CPS kidney gene expression (*R*^2^ < 0.01 for all the genes evaluated). Gray bars indicate the clock time of lights off. *P* < 0.05 Pearson's r.

We evaluated daily transcriptional oscillation in kidneys from LD and CPS fetuses. At E18, we did not observe a 24 h pattern of gene expression in none of the experimental groups. Nevertheless, LD kidney displayed three oscillation peaks repeating approximately every 8 h, with different amplitude for each one of genes evaluated *Bmal1* (R^2^: 0.569, df 31), *Per1* (R^2^: 0.4584, df 24), *Per2* (R^2^: 0.3309, df 22) and *Dbp* (R^2^: 0.4132, df 27) (Figure 1A). The same result was found for the clock controlled-genes *Sgk1* (R^2^: 0.3627, df 27), *aENaC* (R^2^: 0.2386, df 23), *Nhe3* (R^2^: 0.3424, df 23), and Nr3c1 (R^2^: 0.4225, df 26) (Figure 2A). This observation in LD fetuses reveals an early pattern for daily gene oscillation that could be related with developmental maturational changes in the kidney's clock machinery. On the contrary, fetal CPS kidneys showed a flat pattern of expression in the clock genes analyzed (Figure 1B) as well for *Sgk1* (Figure 2B). For α*Enac* and *Nhe3*, the CPS kidneys displayed a non-significant peak at 08:00 and 20:00 h (Figure 2B).

**Figure 3:**
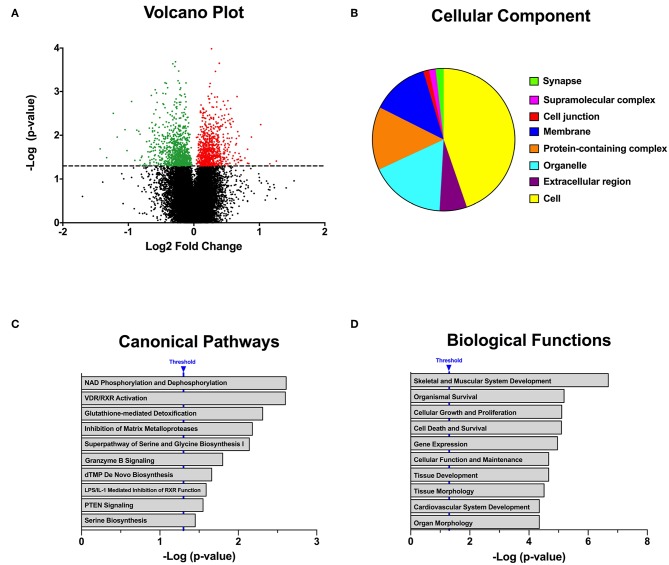
Global transcriptional changes in the fetal kidney from pregnancy under chronodisruption. **(A)** Gestational chronodisruption induces significant differential expression of 1,703 transcripts which are represented using a volcano plot with the up-regulated genes in red and down-regulated genes in green; the segmented line indicates *p*-value 0.05 by One-Way between-subject unpaired ANOVA. **(B)** Pie-chart showing the Cellular Components most represented from gene ontology analysis of the deregulated genes with DAVID. **(C,D)** display functional genomics analysis for Canonical Pathways and Biological Functions impacted by gestational chronodisruption. Blue lines indicate *p*-value 0.05 by right-tailed Fisher's exact test.

Recently, we reported that CPS modifies the transcriptome in an early peripheral clock, namely, the fetal adrenal gland (30). Here, we explored the effects of gestational chronodisruption on global gene expression in fetal kidney at 19 days of gestation. To this end, we compared the LD and CPS fetal transcriptome at 08:00 h clock time, at which corticosterone values in CPS were about half of LD fetuses (30). Using a microarray-based platform accounting for 28,000 genes, we identified 1,703 transcripts that were differentially expressed, as shown in Figure 3A in a volcano plot; with 868 transcripts being down-regulated and 835 being up-regulated in the CPS fetal kidney. To understand the biological significance of this finding, bioinformatics analysis related to gene ontology (GO) classification and pathway analysis was performed to obtain an unbiased view of the CPS effect on fetal kidney gene expression. For this purpose, we used two different bioinformatics tools: DAVID (Database for Annotation, Visualization and Integrated Discovery) and IPA (Ingenuity Pathway Analysis). With DAVID GO analysis the molecular components most represented were Cell function (GO:0005623 43.7%), Organelle (GO:0043226 17.2%), Protein-containing complex (GO:0032991 14.3%) and Membrane (GO:0016020 13%) (Figure 3B). In turn, the most represented cellular compartments were “Plasma Membrane” and “Nucleus,” with 148 and 126 genes, respectively; while for biological functions, “Regulation of transcription” was the most represented with 115 genes. Interestingly, we found that the significant enrichment pathways on KEEG were: “Ribosome” (Rno03010; *p*-value: 5.1E^−4^), “Viral myocarditis” (Rno0516; *p*-value: 4.2E^−2^) and “Aldosterone-regulated Na+ reabsorption” (Rno04960; *p*-value: 4.8E^−2^). On the other hand, for each significant gene, IPA analyzes both fold change and expression effect (up or down), and with this information an integrated analysis is performed against an empirical database. Hence, the impact of CPS conditions on genes related with several “Canonical Pathways” is shown in Figure 3C (where the significant pathways are ordered from minor to major *p*-value). Regarding the “Biological Functions,” they were organized by *p*-value with the lower value in the top in Figure 3D. From this last analysis the activated “Biologicals Functions” (with a z-score > 2) were separated in two groups: related with “Muscle and Bone Development” (differentiation of connective tissue cells, loss of filaments, formation of muscle, bone mineral density, bone differentiation, differentiation of osteoblastic-lineage cells), and “Unspecific Function” as “Transcription of RNA,” “Expression of RNA,” “Quantity of Cells,” “Chemotaxis of Cells” and “Size of Body.” Of note, the inactivated biological functions (with a z-score between −1.9 to −1.8) were related with “Renal and Urological Disease” as the main term; with an inactivation prediction of “Chronic Kidney Disease” as well as “Renal Lesion.” It is important to keep in mind that most of the genes related with pathologies in the software algorithm are related with disease in adult tissue; therefore, the genes involved in normal function or pathologies at fetal developmental stages could be different from those described in the adult kidney.

### Effects of Gestational Chronodisruption in Adult Offspring

Maternal exposure to CPS had no effects on maternal weight nor litter size (29). As described previously, both groups of male offspring showed similar food consumption between weaning and adult age, however CPS offspring were slightly heavier at 90 days of age (LD: 531.3 ± 8.7 g, *n* = 26; vs. CPS: 584.5 ± 10.5, *n* = 32; *P* < 0.05, unpaired *t*-test) (30). However, we did not observe differences in the adult kidney weight (LD: 1.80 ± 0.20 g; CPS: 1.83 ± 0.24 g).

About the long-term effects of CPS, we previously showed that gestational chronodisruption modify the daily rhythms of melatonin, corticosterone, aldosterone and some renal markers in adult offspring (29). Here, we investigated two potential mechanisms by which CPS may induce changes in renal function in the adult offspring, namely, low-grade inflammation and rhythmic gene expression.

**Figure 4:**
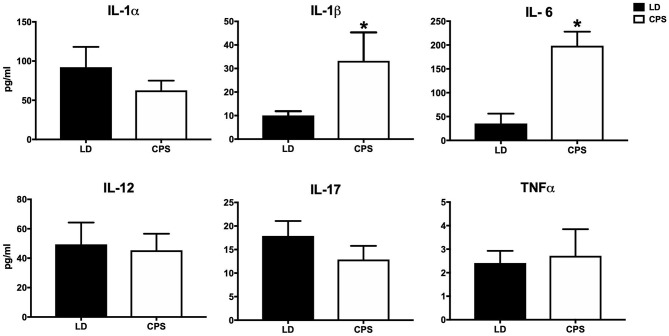
Effect of gestational CPS on circulating cytokines in adult offspring at 90 days of age. Blood samples were collected at 10:00 h (AM; LD *n* = 4 and CPS *n* = 5), a group of selected pro- and anti-inflammatory cytokines were evaluated in the adult plasma. Measurements were carried out using Milliplex RECYTMAG-65K and RECYMAG65K27PMX kits. We did not observe changes in anti-inflammatory cytokines such as IL-10 (LD 73.14 ± 8.86; CPS 68.86 ± 15.12 pg/ml NS). Results are expressed as mean ± SEM. ^*^Different from LD (*P* > 0.05; Mann-Whitney test).

To determine whether gestational chronodisruption could promote a state of low-grade inflammation, me measured some cytokines in the offspring's plasma. The adult CPS displayed a significant increase in two of six proinflammatory cytokines evaluated (Figure 4), whereas no significant difference was found for IL-10 anti-inflammatory cytokine (LD 73.14 ± 8.86; CPS 68.86 ± 15.12, pg/ml, NS). This proinflammatory status could contribute to effects of fetal programming carrying on to the adult offspring.

**Figure 5:**
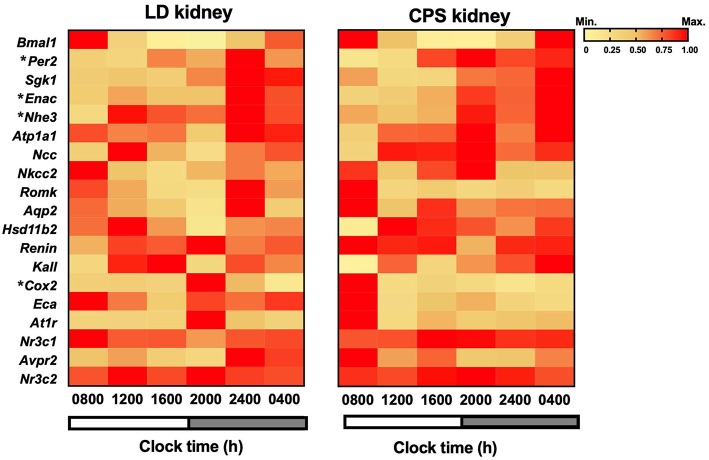
Phase-sorted heat map of whole kidney genes transcribed during a period of 24 h in the adult offspring. **Left**, LD (*n* = 28, *n* = 5/clock time); **Right**, CPS (*n* = 26, *n* = 4/clock time). Individual RNA samples were analyzed using RT-qPCR, and the means were analyzed using a theoretical cosine function. Genes expressed in a circadian manner were plotted on a phase-sorted heat map. Colors indicate min-max normalized relative expression for mean ± SEM of 2-ΔΔCt. Yellow: zero means lowest expression; red: one means maximum expression. Shaded bars represent lights off. ^*^Different from LD (*P* < 0.05; two-way ANOVA and Bonferroni).

Like other organs in the body, the kidney displays circadian fluctuations in gene expression; here we explored the effects of gestational chronodisruption on daily expression of clock genes, transporters, channels, hormone receptors and enzymes. In the adult LD offspring the core-clock genes *Bmal1* and *Per2* presented a daily rhythm in antiphase as described in other adult organs (6) with acrophases times at 22:17 and 14:46 h; respectively. All genes studied, including transporters, channels, enzymes and receptors showed significant daytime differences at the transcriptional level in the kidney from adults gestated under control 12:12 h photoperiod. In turn, gestational CPS conditions slightly affected circadian oscillation of clock genes, with high *Per2* transcript levels at 24:00 h compared to LD; however, a robust daily rhythm was maintained. Regarding other genes, the daily rhythm observed in *Atp1a1, At1r, Nr3c1*, Na-K-Cl cotransporter 2 and arginine vasopressin receptor-2 (*Avpr2*) were all disrupted in the adult CPS kidney (Figure 5). In addition, gestational chronodisruption modified the rhythm and expression level (as a 24 h mean) of *Renin* mRNA (Table 2).

**Table 2:** Statistical parameters for clock-time related changes in kidney mRNA expression (2- ΔΔCt) of LD and CPS adult rats at 90 days of age.

	**LD group**					**CPS group**				
	**ANOVA**		**Cosine wave** **fitting**		**Relative** **expression**	**ANOVA**		**Cosine wave** **fitting**		**Relative** **expression**
	***F***	**Tukey's**	***R^**2**^***	**ϕ**	**24 h mean**	***F***	**Tukey's**	***R^**2**^***	**ϕ**	**24 h mean**
**CLOCK GENES**										
*Bmal1*	F_(5.18)_ = 63.6	08 ≠ 20 h	0.91^*^	22.17 h	0.88 ± 0.69	F_(5.16)_ = 11.2	08 ≠ 20 h^†^	0.69^*^	22.23 h	0.95 ± 0.72
*Per2*	F_(5.20)_ = 7.9	08 ≠ 24 h^†^	0.47^*^	14.46 h	0.65 ± 0.28	F_(5.16)_ = 16.6	08 ≠ 20 h^†^	0.71^*^	13.56 h	0.85 ± 0.54
**TRANSPORTERS AND CHANNELS**										
*Sgk1*	F_(6.25)_ = 10.1	12 ≠ 04 h^†^	0.60^*^	01.38 h	0.99 ± 0.45	F_(6.24)_ = 4.9	12 ≠ 04 h^†^	0.51^*^	00.45 h	0.97 ± 0.52
*αEnac*	F_(6.21)_ = 3.3	n.s.	0.41^*^	02.16 h	0.86 ± 0.21	F_(6.21)_ = 11.4	08 ≠ 20 h^†^	0.60^*^	23.09 h	1.00 ± 0.49
*Nhe3*	F_(6.17)_ = 3.6	08 ≠ 24 h^†^	0.44^*^	21.23 h	0.80 ± 0.19	F_(5.20)_ = 1.5	n.s.	0.54^*^	23.35 h	0.97 ± 0.28
*Atp1a1*	F_(5.18)_ = 7.6	08 ≠ 20 h^†^	0.31^*^	20.34 h	2.47 ± 0.62	F_(5.17)_ = 2.7	n.s.	0.24	n.s.	2.03 ± 0.75
*Ncc*	F_(5.16)_ = 12.9	12 ≠ 22 h^†^	0.04	n.s.	0.83 ± 0.4	F_(5.15)_ = 2.8	08 ≠ 12 h^†^	0.31	n.s.	1.38 ± 0.38
*Nkcc2*	F_(5.19)_ = 2.5	08 ≠ 16 h^†^	0.28	n.s.	1.04 ± 0.45	F_(5.15)_ = 2.7	n.s.	0.02	n.s.	1.09 ± 0.36
*Romk*	F_(6.20)_ = 1.6	n.s.	0.21	n.s.	1.35 ± 0.68	F_(6.20)_ = 2.9	n.s.	0.21	n.s.	1.17 ± 0.47
*Aqp2*	F_(6.19)_ = 1.2	n.s.	0.15	n.s.	1.09 ± 0.50	F_(6.19)_ = 0.8	n.s.	0.01	n.s.	1.12 ± 0.29
**ENZYMES**										
*Hsd11b2*	F_(5.18)_ = 2.3	12 ≠ 20 h^†^	0.24	n.s.	1.21 ± 0.54	F_(5.16)_ = 6.2	08 ≠ 20 h^†^	0.16	n.s.	1.69 ± 0.77
*Renin*	F_(5.22)_ = 3.1	08 ≠ 20 h^†^	0.25	n.s.	0.74 ± 0.17	F_(5.17)_ = 0.9	n.s.	0.12	n.s.	1.03 ± 0.19^‡^
*Kall*	F_(6.20)_ = 3.7	n.s.	0.08	n.s.	0.65 ± 0.30	F_(6.20)_ = 11.9	08 ≠ 24 h^†^	0.27	n.s.	0.78± 0.59
*Cox-2*	F_(6.22)_ = 7.2	08 ≠ 20 h^†^	0.26	n.s.	1.13 ± 0.71	F_(6.20)_ = 3.9	n.s.	0.23	n.s.	1.35 ± 0.74
*Eca*	F_(6.22)_ = 3.5	08 ≠ 16 h^†^	0.33	n.s.	1.33 ± 0.61	F_(6.20)_ = 4.5	08 ≠ 24 h^†^	0.13	n.s.	1.6 ± 0.86
**RECEPTORS**										
*At1r*	F_(5.20)_ = 9.2	08 ≠ 20 h^†^	0.37^*^	12.49 h	0.74 ± 0.40	F_(5.16)_ = 2.4	08 ≠ 12 h^†^	0.19	n.s.	0.71 ± 0.33
*Nr3c1*	F_(5.20)_ = 4.7	08 ≠ 20 h^†^	0.43^*^	13.09 h	0.92 ± 0.16	F_(5.18)_ = 1.0	n.s.	0.13	n.s.	0.96 ± 0.10
*Avpr2*	F_(6.21)_ = 1.8	n.s.	0.34^*^	04.04 h	1.35 ± 0.45	F_(6.22)_ = 0.1	n.s.	0.00	n.s.	1.40 ± 0.16
*Nr3c2*	F_(5.20)_ = 3.6	n.s.	0.16	n.s.	0.84 ± 0.09	F_(5.16)_ = 0.8	n.s.	0.09	n.s.	0.95 ± 0.08

n.s., not significant;

* *, Significant fitting to cosine curve; P < 0.05 Pearson's r*.

† *, significant differences from other clock times; P < 0.05 Tukey's*.

‡ *, different from LD, P < 0.05. un paired t-test*.

*ϕ, clock time at acrophases*.

### Blood Pressure in Adult Offspring

We determined the systolic blood pressure (SBP) at 12:00 and 20:00 h to confirm the differences of SBP previously reported by us at 90 days of age (29). Here, we confirmed that the main BP differences between LD and CPS offspring appear during active phase at 20:00 h (data not shown) and therefore we evaluated the response of BP to 4% NaCl at this time point (i.e., where the CPS animals display higher blood pressure than LD animals).

**Figure 6:**
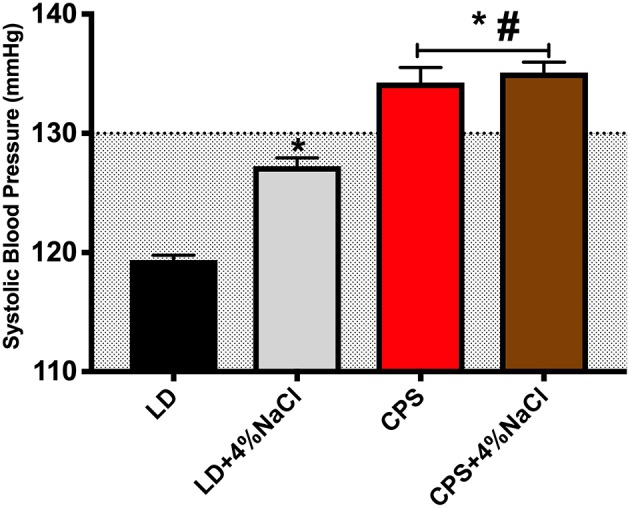
Systolic blood pressure in response to 4%NaCl. Adult males were evaluated at 150 days of age (LD *n* = 8; CPS *n* = 7). The animals were trained 7 days before basal records. SBP was evaluated at 20 h by tail cuff method. Measure is the average for five records, actual data are mean ± SEM. ^*^Different from LD, ^**#**^Different from LD+4%NaCl (*P* < 0.05 One way ANOVA and Tukey's). The gray area indicates the normal range of SBP for the rat.

Next, we assessed SBP in four groups of animals that were studied in parallel, LD and CPS males with standard chow (normal sodium; 0.26% NaCl) and LD+4%NaCl and CPS+4%NaCl that were challenged by overload of sodium in the food. As we expected, the LD and CPS animals maintained SBP values (LD 122 ± 0.31; CPS 134.3 ± 8.90 mmHg) close to our previous findings reported in Mendez et al. (29). When we compared the four groups of animals at the end of the protocol (at 150 days of age), the record obtained in LD and LD+4%NaCl remained inside the normal range for the species, however the CPS and CPS+ 4%NaCl animals maintained elevated levels of BP (about 135 mmHg) (Figure 6). On the other hand, we obtained urine using metabolic cages for 16 h, to analyze markers of renal function at basal time and at the end of 4 weeks of the 4% NaCl treatment. At basal time we observed higher levels of proteins (LD 3.63 ± 0.70; CPS 5.87 ± 0.39^*^ mg/dl/16 h; Mann-Whitney test and sodium (LD 0.45 ± 0.05; CPS 0.75 ± 0.09^*^ mEq/l/16 h Mann-Whitney test) in urine in adult offspring gestated under CPS conditions. At the end of protocol, the adult CPS+4% NaCl animals displayed a significant increase in creatinine, sodium and chloride. We also analyzed the presence of pathological markers finding changes in eight of them. These results highlight that gestational chronodisruption triggers negative modifications in the adult CPS offspring, given that the markers evaluated here are used for detection of kidney damage in different models of renal injury (Table 3).

**Table 3:** Urinary markers in adult offspring gestated in LD and CPS conditions, and challenged with 4%NaCl for 4 weeks.

	**Normal sodium (0.23%)**		**High sodium (4%)**	
	**LD**	**CPS**	**LD +4% NaCl**	**CPS +4% NaCl**
**URINE 16-h**				
Creatinine(mg/dl)	7.95 ± 0.40	6.77 ± 0.47	4.45 ± 0.53	6.14 ± 0.50^#^
Proteins (mg/dl)	2.58 ± 0.43	6.56 ± 0.87^*^	1.22 ± 0.18	2.47 ± 0.36
Na (mEq/L)	0.45 ± 0.05	0.75 ± 0.09	4.48 ± 0.82^*^	7.17 ± 0.92^#^
K (mEq/L)	1.68 ± 0.13	2.08 ± 0.16	1.23 ± 0.20	2.23 ± 0.18
Cl-(mEq/L)	1.22 ± 0.09	1.24 ± 0.10	4.24 ± 0.76^*^	6.70 ± 0.82^#^
**PATHOLOGICAL MARKERS (ng/ml/16h)**				
Alb	403.2 ± 73.48	1145 ± 517	589.3 ± 214	993.1 ± 353.2
AGP	44.91 ± 5.37	36.34 ± 6.11	33.00 ± 5.79	42.01 ± 4.98
Clusterin	5090 ± 1022	1996 ± 848^*^	3213 ± 287	1503 ± 690.9^*^
GST	45.62 ± 8.13	144.10 ± 63.37^#^	21.75 ± 3.75	48.41 ± 10.73^#^
Kim1	1.50 ± 0.54	14.02 ± 4.23^*^^#^	1.34 ± 0.13	21.07 ± 7.45^*^^#^
OPN	0.25 ± 0.9	13.00 ± 4.70^*^^#^	0.23 ± 0.07	20.4 ± 7.70^*^^#^
Tim1	240.9 ± 78.43	79.69 ± 27.07	158.6 ± 36.56	107.2 ± 54.05
VEGF	5.69 ± 1.51	14.95 ± 4.00^*^^#^	4.92 ± 0.56	28.34 ± 6.15^*^^#^
B2M	555.7 ± 106.0	288.4 ± 155.5	483.1 ± 86.09	393.6 ± 143.3
Cystatin	7.60 ± 1.25	17.75 ± 3.70^*^	9.73 ± 1.27	28.64 ± 6.06^*^^#^
EGF	3.80 ± 0.62	16.34 ± 3.35^*^	10.75 ± 1.37^*^	26.82 ± 5.56^*^^#^
Lipocalin	1.90 ± 0.31	13.09 ± 4.46^*^	2.71 ± 0.45^*^	21.28 ± 7.42^*^
Calbindin	1758 ± 1154	264.8 ± 75.58	1245 ± 592.2	332.7 ± 159.9
IP-10	0.29 ± 0.12	12.39 ± 4.74^*^	0.83 ± 0.18	20.61 ± 7.62^*^^#^

Concentrations of electrolytes, creatinine, proteins and pathological markers and were adjusted by urine volume obtained in 16-h

* *Different from LD*,

# *Different from LD +4% NaCl; p < 0.05 Kruskal-Wallis rank test corrected by False Discovery Rate. (Two-stage step-up method of Benjamini, Krieger and Yekutieli)*.

**Figure 7:**
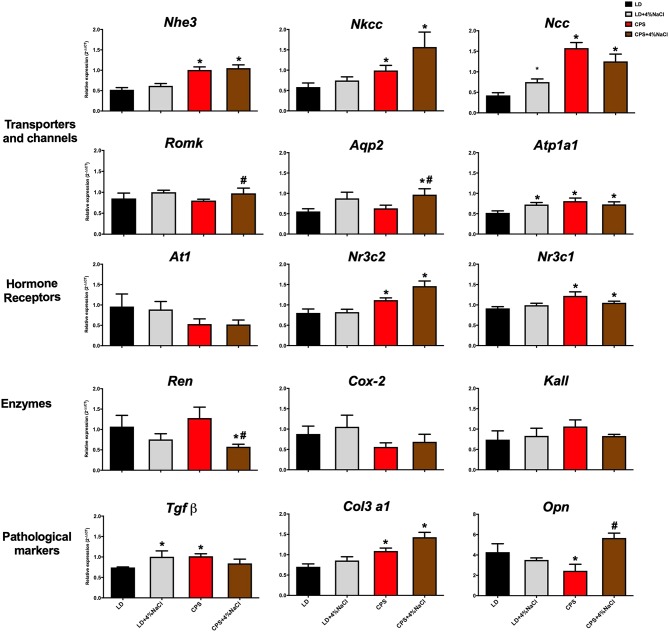
Gene expression in response to 4%NaCl in adult offspring gestated under LD and CPS conditions. Whole kidneys were obtained at 150 days of age (LD *n* = 8; CPS *n* = 7) at 10:00 h for the study of gene expression in males gestated under LD and LD+4%NaCl conditions (black and gray bars; respectively); CPS and CPS+4%NaCl (red and brown bars; respectively). Data are mean ± SEM ^*^Different from LD; ^#^Different from CPS (*P* < 0.05 Kruskal-Wallis test).

To further understand the impact of gestational CPS treatment on blood pressure regulation, relative expression of key genes related with kidney function was evaluated in LD, LD+4%NaCl, CPS and CPS+4%NaCl. Gestational chronodisruption programmed relative expression of transporters, hormone receptors and pathological markers. As shown in Figure 7, *Nhe3, Ncc, Nkcc2, Atp1a1, Nr3c1*, and *Nr3c2* mRNA levels were modified by CPS; meanwhile genes as *Nhe3, Nkcc2, Ncc*, and *Nr3c2* were sensitive to 4 weeks high-sodium diet. Finally, the pathological markers *Tgf*β, *Col3a1* and *Opn* were modified in adults gestated under CPS conditions, while *Col3a1* and *Opn* increased in response to 4% NaCl. Collectively, these results suggest that altered blood pressure in the adult CPS offspring is strongly related with kidney dysfunction.

**Figure 8:**
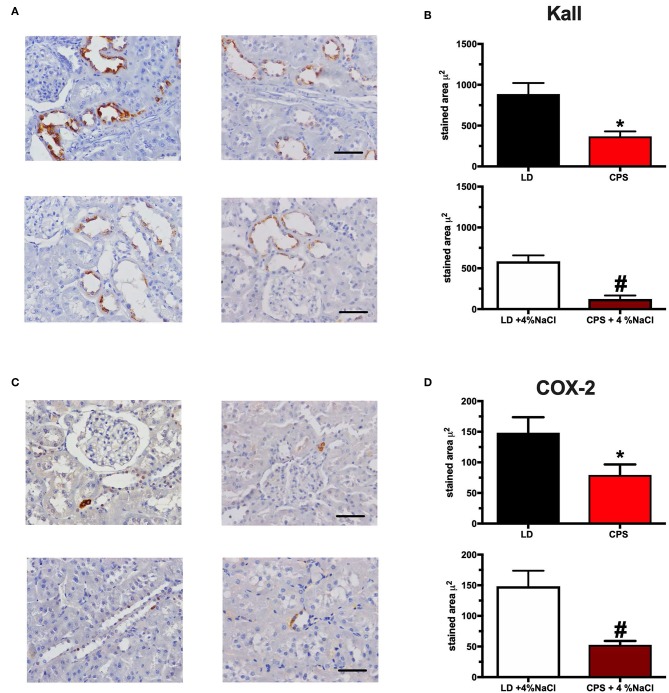
Protein expression of Kallikrein and COX-2 in adult males gestated under LD and CPS conditions. **(A)**. Representative sections for Kallikrein immunostaining in renal tissue from LD, CPS, LD +4% NaCl and CPS+4%NaCl. 40X objective, 100 μm scale. **(B)** Whole data analysis for staining area for Kallikrein in renal tissue from adult males. ^*^Different from LD; ^#^Different from LD+4%NaCl (*p* < 0.05, Student's *t*-test). **(C)** Representative sections for COX-2 immunostaining in renal tissue from LD, CPS, LD +4% NaCl, and CPS+4%NaCl. 40X objective, 100 μm scale. **(D)** Whole data analysis for staining area for staining area for COX-2 in renal tissue from adult males. ^*^Different from LD; ^#^Different from LD+4%NaCl (*p* < 0.05, Student's *t*-test). Values are mean ± SEM.

Together with the analyses of relative gene transcription, we studied the effects of sodium on the morphology of the kidneys. At 150 days of age, we observed no evident morphological differences between the four groups studied. Besides, Kallikrein and COX-2 proteins were analyzed; both proteins are involved in the regulation of sodium in the thick loop of Henle and in the collecting duct, respectively. Regarding the expression of Kallikrein, we observed that CPS offspring showed a lower staining area with respect to LD; while increased sodium intake for 4 weeks decreased the expression of Kallikrein in both groups, LD+4%NaCl and CPS+4%NaCl (Figure 8). A similar result was observed for COX-2 protein. Therefore, gestational chronodisruption decreased the expression of COX-2 in the adult kidney, while 4 weeks of 4%NaCl treatment decreased the expression of the enzyme in both LD+4%NaCl and CPS+4%NaCl offspring.

## Discussion

Intrauterine conditions in which the fetus develops have an important influence on the phenotype expressed in adult life. The long-term effects in different models of fetal programming include alterations in gene expression and organ-specific changes, demonstrating an association between environmental and epigenetic influences on the outcome of fetal programming (34, 35). Over the last few years, it has been suggested that circadian disruption is a suboptimal condition during gestation that may lead to late-onset of cardiovascular and metabolic diseases. In this context, there is growing evidence indicating that gestational chronodisruption has adverse effects on the mother, the fetus and the adult offspring in some animal models (29, 36–41). These reports support the idea that gestational chronodisruption disturbs fetal and maternal physiology, acting as an adverse intrauterine condition that programs the adult health and impose significant long-term trade-offs. Previously, we showed that adult rats gestated under CPS conditions display changes in daily rhythms of melatonin, corticosterone, aldosterone, some renal markers, featuring modified blood pressure and heart rate at 90 days of age (29). Given that circadian rhythms in various renal functions and blood pressure have been described (42, 43), here we investigated whether chronic photoperiod shift during gestation (CPS—chronic photoperiod shifting; up to 85% of gestation), modifies the kidney biological clock and blood pressure control in the adult offspring.

### Effects of Gestational Chronodisruption on the Fetal Kidney Clock

The present study is the first demonstration of fetal programming of adult kidney dysfunction and prehypertensive phenotype by gestational chronodisruption. One limitation of our results is the use of non-sexed fetal kidneys for RT-qPCR and microarray, which was not included in the original design of the experiments. Therefore, the possibility of long-term sex-differences in the outcomes studied here needs to be addressed.

The core components of the clock machinery are expressed early in several fetal tissues, such as liver, kidney and adrenal gland (44, 45). In fact, the evidence available demonstrates that the circadian clock begins ticking early in life, and therefore, it may be influenced by maternal signals or by the mother's external environment; as it has been observed in several models of gestational chronodisruption (29, 30, 37, 38, 40, 46). In a rat model, we have demonstrated that the fetal adrenal clock is ticking during gestation and that CPS has adverse effects on this peripheral clock at 18 days of gestation (30). Here we studied the potential early oscillation of the kidney clock at E18 and the effects of gestational CPS on renal gene expression.

At E18, we did not find a 24-h oscillation of clock genes nor clock-controlled genes. This result agrees well with data reported in mice embryos, where no circadian rhythmicity in clock genes was observed in mouse liver, kidney and heart at E18 (47). However, here we observed an interesting pattern of gene oscillation that is recurrent close to 8-h in almost all genes studied in the fetuses gestated in LD conditions. This pattern of oscillation could be a preparation for the future circadian transcription in the kidney clock, whose period and amplitude would vary according to developmental changes. In fact, circadian amplitude displays changes in several organs indicating that the temporal order of physiological functions varies during development and reflect organ-specific entrainment at different developmental stages (44, 45, 48, 49).

Of note, an early 24 h oscillation of *Per2* and *Rev-erba* (as well as the clock-controlled genes α*Enac, Sgk1*, and *Nhe3*) was demonstrated in the rat fetal kidney at E20 (28); in contrast with our finding of 8-h oscillatory gene expression patterns for clock and clock-controlled genes at E18. This discrepancy might be explained by the fact that there are marked developmental differences between 18 and 20 days of gestation (term at 21 days), a time window where exponential and maximal fetal growth has been described in the rat (50). In this unique physiological context, it is conceivable that kidney maturation along the late phase of rat pregnancy undergoes dramatic changes, so that short-term differences in gene expression patterns may be somewhat predictable. Interestingly, here we observed that gestational chronodisruption abolished this rhythmicity-like pattern in the CPS kidney; demonstrating effects of gestational circadian disruption on the fetal kidney at E18. Although there are significant age and interspecies differences in the timing of gene oscillation, proper initiation of circadian rhythms (associated with peripheral clock activation) could be an integral aspect of the developmental process. Therefore, the acquisition of biological rhythms correctly synchronized could be a determinant of fetal development, eventually laying the ground for appropriate transition to adulthood.

As we reported previously, circadian disruption impacts the fetal transcriptome of heart, liver and adrenal gland in different models of gestational chronodisruption (30–32). Our microarray studies in fetal kidney revealed that 1,703 transcripts were differentially expressed, where the most represented Cellular Compartment were “Plasma Membrane” and “Nucleus,” while the main Biological Function targeted was “Regulation of transcription.” Also, we found that the significant enrichment pathways on KEEG were: “Ribosome,” “Viral myocarditis” and “Aldosterone-regulated Na+ reabsorption.” Many of the differentially expressed genes in the CPS kidney have high representation during the embryonic developmental stage; for instance, cellular proliferation, morphogenesis and ECM production, as it has been described in ontogenic kidney array studies (51, 52). However, the inactivated biological functions identified were related with the main term “Renal and Urological Disease” with an inactivation of “Chronic Kidney Disease” and “Renal Lesion.” A plausible explanation for this is that the software algorithm works with a database related with disease in the adult kidney, whereby this prediction of “protection” could be the representation of the normal changes occurring during growth, where an important number of genes are expressed differently during fetal and adult life. This possibility has been showed in studies comparing fetal and adult stages, where embryonic kidney is committed to cellular proliferation and morphogenesis early on, followed by extracellular matrix deposition and acquisition of markers of terminal differentiation with the expression of transporters, detoxification enzymes and oxidative stress genes (51, 52). To confirm our results and also to extend the present microarray-predicted expression, we validated the study using RT-qPCR, where genes as *Col6A* and *Col1A* (involved in ECM deposition) were validated (data not shown). Although not all genes were statistically validated (probably by different limit of detection of both methods), all genes included in the validation showed the same results (up-down regulation) relative to the microarray results. With the exception of “Aldosterone-regulated Na+ reabsorption,” we did not identify more pathways related directly with kidney function. However, we believe that the modification of 1,703 transcripts by CPS could have a significant integrated impact on the fetal kidney, considering that the tissue was evaluated in a period characterized by developmental changes, which actually ends up only in the postnatal period in the rat; hence, it is conceivable that although immature the kidney can be programmed by gestational chronodisruption at 18 day of gestation.

### Effects of Gestational Chronodisruption on Adult Offspring

There is evidence suggesting that insults taking place as early as during gestation could lead to elevated blood pressure in the adult offspring. However, the precise grounds for this BP increase remain unknown. Epigenetic modifications have been proposed as a probable mechanism. For example, using a rodent model of maternal protein restriction during gestation, a decrease in the methylation at CpG dinucleotide has been observed at the proximal promoter region of the angiotensin receptor (53). Recently, we demonstrated that gestational chronodisruption modified global DNA methylation in the CPS adrenal gland at 90 Days of age (30). We also found decreased global DNA methylation in the kidney from CPS offspring relative to adults gestated under standard 12:12 h photoperiod (unpublished data). It is possible that the absence of melatonin in the adult CPS (29) may play a role as an epigenetic modifier contributing to elevated BP in the CPS offspring (54). Besides, melatonin is required to maintain the phase relationship between the SCN and peripheral clocks, also exerting a myriad of functions; such as modulation of the inflammation processes and strong antioxidant activity, among others (55) therefore, suppression of melatonin may trigger multiple changes in peripheral clocks as the kidney, with effects on blood pressure.

One potential mechanism promoting hypertension is inflammation. Circadian disruption has been related with an increase of inflammatory cytokines contributing to risk of cardiovascular disease (56). Here we evaluated the presence of some features of low-grade inflammation in adults gestated under gestational chronodisruption and found an increase of pro-inflammatory cytokines IL-1β and IL-6 in adult CPS animals. The relevance of increased circulating pro-inflammatory cytokines becomes apparent considering that they activate signal transduction cascades impinging on transcriptional activity in different target cells. To the best of knowledge, there is no reported evidence of fetal programming of adult pro-inflammatory status triggered by altered photoperiod along gestation. However, epidemiological and experimental lines of evidence strongly suggest that different prenatal adverse conditions may translate into adult immune disease (57). These authors state that other than orchestrating local and systemic responses, pro-inflammatory cytokines activate the HPA axis contributing to a rise of glucocorticoids levels, which in turn suppresses further cytokine production (57). On the other hand, the present results do not rule out that the increase of IL-1β and IL-6 observed in the adult CPS offspring may derive from altered gene expression patterns in the kidney (present report) and/or effects on some components of the endocrine system; for instance, the absence of daily rhythm of plasma corticosterone and aldosterone, together with an increase of circulating corticosterone (29, 30). At any rate, systemic inflammation participates in several steps in the development of cardiovascular disease, including endothelial dysfunction and oxidative stress; in fact, presently it can be considered an independent risk factor for essential hypertension (56, 58). Taken together, our results suggest that gestational chronodisruption could promote a systemic low-grade inflammation state, which, together with our previously reported changes in circulating hormones (29), might contribute to the increase of BP in the adult CPS offspring.

It is known that kidney physiology is characterized by a periodicity of 24-h for gene expression, contributing to various cardiovascular functions (26, 59). Indeed, some genes such as *Nhe3* and α*ENaC* display E-Box motifs in their promoter regions, which are regulated directly by clock genes, demonstrating the role of renal clock in handling sodium, chloride, potassium and water and therefore participating in the control of BP (26, 59–62). Many studies show that disrupted rhythmic activity is a phenotype often seen in numerous cardiovascular diseases (24, 63), where alterations on sympathetic vascular tone and renal sodium handling could be involved and, interestingly, these functions are under circadian control (24). A small number of studies have examined aspects like tubular handling of sodium in fetal programming. Alterations of some components at the tubular level, such as of *Nkcc2* and *Ncc* have been reported, suggesting that renal handling of sodium contributes to arterial hypertension in the adult offspring, as shown in a classical model of protein restriction during gestation (64). As expected, we observed that adult LD offspring showed circadian oscillation in clock and clock-controlled genes as documented in whole rodent kidney (24, 28); the relative expression level of many of the mRNAs was disrupted in the CPS kidney, as well as the 24 h mean expression level of *Renin* mRNA. Since functional genes were disrupted in the CPS offspring's kidney (despite an apparent kidney clock integrity), our results indicate a general compromise of kidney function that could translate into changes of key functions, such as blood pressure control.

As mentioned, suboptimal intrauterine environment may modify the normal developmental trajectory and thus have potential long-lasting effects. However, postnatal development and adult behavioral factors modify the progression of these alterations. The kidney plays a key role in the generation of hypertension and controls BP through regulation of extracellular volume (balance in the urinary excretion of sodium and water), therefore dysfunctional sodium excretion increases the susceptibility to hypertension (22, 23). Here we subjected the adult CPS offspring to a 4%NaCl challenge for 4 weeks. Although this challenge did not trigger hypertension in adult CPS animals, their blood pressure remained high at the end of 4 weeks. Besides, both groups (CPS and CPS+4%NaCl) analyzed in the study display changes in several pathological markers, remarking the effects of gestational chronodisruption.

The control of fluids is an important mechanism used to regulate blood pressure. As extracellular fluid volume increases in response to high salt intake, the kidneys excrete the excess fluid and electrolytes in order to return the extracellular fluid volume and BP to normal levels. In CPS animals, despite the attempt to eliminate more sodium, the SBP remained elevated (close to 145 mmHg), which may be characterized as a prehypertensive state.

The blood pressure response to sodium overload is an age-dependent process in rats and it coincides with the phase of higher growth, occurring between 4 and 10 weeks old. Puberty and sexual maturation are a time of profound hemodynamic adjustments, such as decreased cardiac output and increased vascular resistance, resulting in hemodynamic maturity that stabilizes BP levels in adults (65). In CPS offspring we know that several endocrine changes occur, as modifications in daily rhythm of melatonin and corticosterone (29, 30). In the kidney, glucocorticoids help to regulate body fluid homeostasis by improving the diuretic and natriuretic response in the inner medullary-collecting duct and can regulate circadian genes in the kidney (26). For example, Ivy et al. (66) found that chronic corticosterone infusion led to increased levels of *pNCC, Bmal1* and *Per1* during the inactive phase. Besides, adrenalectomy resulted in a blunted rhythm of *pNCC* expression in the kidney (66). Again, the absence of circulating melatonin could contribute to the phenotype observed in adult CPS. In fact, pinealectomy in rats (and consequent suppression of melatonin) induces a moderate elevation of BP in prepubertal males, and slight changes in adult males. This prehypertensive state culminates in hypertension after administering a dietary sodium overload (67, 68).

Together with changes in SBP we observed modification of the expression level of several genes after sodium intake (4 weeks) in the adult CPS offspring. In this context, it has been reported that the consumption of high sodium in the diet in mice was able to modify the rate and timing of gene expression in the kidney (69). It is likely that the 4% NaCl treatment could have a greater impact on CPS kidneys, which already display changes in gene expression in several segments of the nephron. About 90-95% of the sodium is reabsorbed in the proximal portion of the nephron; however, the distal nephron plays a crucial role in sodium handling (70). The Kallikrein-kinin system (KKS) is a multienzyme system including the enzyme kallikrein, their substrates (high and low molecular weight kininogens) and vasoactive hormones formed (bradykinin); whose activation produces a wide spectrum of biological effects, including vasodilation, diuresis and natriuresis. Genetically hypertensive rats show decreased excretion of urinary kallikrein, while this condition is an indicator of salt sensitivity in humans and rats (71–73). The susceptibility of changes in the BP to excessive sodium intake has been evaluated in different experimental models, suggesting that defects in the KKS could sensitize to significant cardiovascular effects after sodium overload (73). Here we studied the effects of CPS on Kallikrein and COX-2. Gestational chronodisruption decreased the expression of Kallikrein in CPS animals. Kallikrein expression and distribution is highly dependent on the development process, thus perinatal disorders such as gestational chronodisruption may contribute to the programming of this system. Another possibility is that changes in Kallikrein may be linked to adaptation to changes in gene expression or aldosterone changes (29). In this regard, adrenalectomized rats excrete less than half of Kallikrein than sham-operated, similarly to administration of aldosterone antagonists; while dexamethasone increases production of Kallikrein (70, 74).

Intake of 4%NaCl decreased expression of Kallikrein in both experimental groups, a situation that has been observed in different models with high sodium intake and in contrast, sodium restriction increases the excretion of Kallikrein in urine (70). The results on CPS+4%NaCl offspring indicate a greater susceptibility of these animals to hypertension and its secondary consequences. A similar situation was observed for the expression of COX-2 protein. Cyclooxygenase (COX-1 and COX-2) are enzymes that represent the rate limiting step for the synthesis of prostaglandins, which are important mediators of renal vascular tone and sodium balance. Although COX-2 has been classified as an inducible enzyme (unlike COX-1, which is “constitutive”), COX-2 is also constitutively expressed in the kidney (75). The specific action of the metabolites of COX-2 is determined by the stringent localization in its expression, such as in macula densa and in the thick ascending loop of Henle, consistent with a role of COX-2 in regulating tubule-glomerular feedback through the release of prostaglandin E2 (PGE2) (76, 77). As Kallikrein, sodium restriction in the diet increases the expression of COX-2, while a high sodium intake leads to a decrease in the expression level; besides its inhibition causes an elevation of blood pressure in rats (78).

The alteration of Kallikrein and COX-2 gene expression in the adult kidney, imposed by gestational CPS conditions, suggests fetal programming of renal function associated with increased susceptibility of the adult offspring to display cardiovascular disorders such as hypertension. Other factors involved in the programming of renal function in the CPS offspring, such as increases in pro-inflammatory cytokines and dysregulation in the expression of several key genes may all contribute to high blood pressure in adult animals. It is also worth emphasizing that high blood pressure was found in the adult CPS offspring under high-salt diet conditions, which is in line with epidemiological evidence consistently reported in humans consuming high-salt diets. In conclusion, gestational chronodisruption imposes persistent alterations of renal function at different levels, which together with the endocrine changes previously reported by us in the adult CPS offspring, might be responsible for the prehypertensive phenotype observed in adult rats that had been gestated under conditions of chronic photoperiod shifting, akin night shift work in humans.

## Data Availability

This manuscript contains previously unpublished data. The name of the repository and accession number are Gene Expression Omnibus (NCBI) and GSE130744; respectively.

## Ethics Statement

The protocols were approved by the Bioethics Commission from the Universidad Austral de Chile (CBA: 221/2015; 297/2017). Animal handling was performed following the guidelines for the care and use of Laboratory Animals of the Institute for Laboratory Animals Research of the National Research Council.

## Author Contributions

NM, CT-F, and HR conceived and designed the study, analyzed and interpreted the data, drafted the manuscript, critically revised important intellectual content in the manuscript, and provided overall supervision. NM, CS, ES, PB, CB, KV, and DH, performed the experiments and analyzed the data and drafted the manuscript, and contributed to intellectual content in the manuscript. CV analyzed and interpreted the data and contributed to intellectual content in the manuscript. All authors approved the final manuscripts and agreed to be accountable for all aspects of the work.

### Conflict of Interest Statement

The authors declare that the research was conducted in the absence of any commercial or financial relationships that could be construed as a potential conflict of interest.
