# Supplementary figures and images for: Fetal Programming of Renal Dysfunction and High Blood Pressure by Chronodisruption
Source: Front Endocrinol (Lausanne). 2019 Jun 6;10:362. doi: 10.3389/fendo.2019.00362 (PMC6563621; doi:10.3389/fendo.2019.00362)

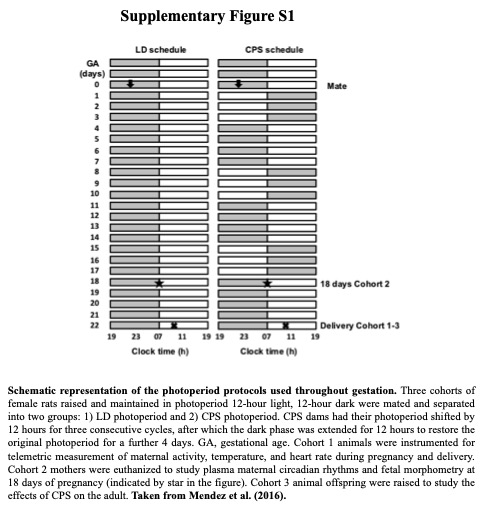

Supplement: Supplementary file 1 [file Image_1.JPEG]
